# Development of GBRT Model as a Novel and Robust Mathematical Model to Predict and Optimize the Solubility of Decitabine as an Anti-Cancer Drug

**DOI:** 10.3390/molecules27175676

**Published:** 2022-09-02

**Authors:** Walid Kamal Abdelbasset, Shereen H. Elsayed, Sameer Alshehri, Bader Huwaimel, Ahmed Alobaida, Amal M. Alsubaiyel, Abdulsalam A. Alqahtani, Mohamed A. El Hamd, Kumar Venkatesan, Kareem M. AboRas, Mohammed A. S. Abourehab

**Affiliations:** 1 Department of Health and Rehabilitation Sciences, College of Applied Medical Sciences, Prince Sattam bin Abdulaziz University, P.O. Box 173, Al-Kharj 11942, Saudi Arabia; 2 Department of Physical Therapy, Kasr Al-Aini Hospital, Cairo University, Giza 12613, Egypt; 3 Department of Rehabilitation Sciences, College of Health and Rehabilitation Sciences, Princess Nourah bint Abdulrahman University, P.O. Box 84428, Riyadh 11671, Saudi Arabia; 4 Department of Pharmaceutics and Industrial Pharmacy, College of Pharmacy, Taif University, P.O. Box 11099, Taif 21944, Saudi Arabia; 5 Department of Pharmaceutical Chemistry, College of Pharmacy, University of Hail, Hail 81442, Saudi Arabia; 6 Department of Pharmaceutics, College of Pharmacy, University of Hail, Hail 81442, Saudi Arabia; 7 Department of Pharmaceutics, College of Pharmacy, Qassim University, Buraidah 52571, Saudi Arabia; 8 Department of Pharmaceutics, College of Pharmacy, Najran University, Najran 11001, Saudi Arabia; 9 Department of Pharmaceutical Sciences, College of Pharmacy, Shaqra University, Al Dwadmi 11961, Saudi Arabia; 10 Department of Pharmaceutical Analytical Chemistry, Faculty of Pharmacy, South Valley University, Qena 83523, Egypt; 11 Department of Pharmaceutical Chemistry, College of Pharmacy, King Khalid University, Abha 62529, Saudi Arabia; 12 Department of Electrical Power and Machines, Faculty of Engineering, Alexandria University, Alexandria 21928, Egypt; 13 Department of Pharmaceutics, College of Pharmacy, Umm Al-Qura University, Makkah 21955, Saudi Arabia; 14 Department of Pharmaceutics and Industrial Pharmacy, Faculty of Pharmacy, Minia University, Minia 61519, Egypt

**Keywords:** optimization, anti-cancer drug, simulation, artificial intelligence

## Abstract

The efficient production of solid-dosage oral formulations using eco-friendly supercritical solvents is known as a breakthrough technology towards developing cost-effective therapeutic drugs. Drug solubility is a significant parameter which must be measured before designing the process. Decitabine belongs to the antimetabolite class of chemotherapy agents applied for the treatment of patients with myelodysplastic syndrome (MDS). In recent years, the prediction of drug solubility by applying mathematical models through artificial intelligence (AI) has become known as an interesting topic due to the high cost of experimental investigations. The purpose of this study is to develop various machine-learning-based models to estimate the optimum solubility of the anti-cancer drug decitabine, to evaluate the effects of pressure and temperature on it. To make models on a small dataset in this research, we used three ensemble methods, Random Forest (RFR), Extra Tree (ETR), and Gradient Boosted Regression Trees (GBRT). Different configurations were tested, and optimal hyper-parameters were found. Then, the final models were assessed using standard metrics. RFR, ETR, and GBRT had R2 scores of 0.925, 0.999, and 0.999, respectively. Furthermore, the MAPE metric error rates were 1.423 × 10^−1^ 7.573 × 10^−2^, and 7.119 × 10^−2^, respectively. According to these facts, GBRT was considered as the primary model in this paper. Using this method, the optimal amounts are calculated as: P = 380.88 bar, T = 333.01 K, Y = 0.001073.

## 1. Introduction

Recent studies in the area of clinical pharmacology have necessitated the invention of novel, promising, and environmentally friendly tools to increase the performance of therapeutic drugs [1,2]. In order to achieve this purpose, numerous efforts have been made to develop disparate approaches to reduce the application of potentially detrimental/deleterious organic solvents.

Decitabine (currently sold under the brand name DACOGEN^®^) is an intravenously administered chemotherapeutic drug, which acts as a nucleoside metabolic inhibitor [3,4,5]. Despite the emergence of some adverse events such as neutropenia, thrombocytopenia, and embryo–fetal toxicity in patients, the drug’s great efficiency in ameliorating MDS has encouraged researchers to use it extensively [6,7,8]. Having long been considered, the most important duty of research and development (R&D) centers of pharmaceutical companies has been to concentrate on the development of supercritical fluid technology (SCFT) [9].

The existence of noteworthy advantages such as negligible processing times, the manufacturing of no organic co-solvent, and its great capability of extracting bioactive molecules has encouraged the researchers to use SCFT for drug discovery from natural sources [10,11,12]. In recent years, CO_2_ supercritical fluid (CO_2_SCF) has received more attention within SCFT as an efficient solvent, due to significant privileges such as chemical inactivity, availability, cost-effectiveness, low critical temperature/pressure, and its approval as a food-grade solvent [13].

In recent years, artificial intelligence (AI) has found its place as a versatile tool, offering a high potential for applications in different industries such as separation, extraction, and nanotechnology, as well as drug development, including the identification, validation, and designation of novel drugs [14,15]. The various advantages of AI technology, such as robustness and time-effectiveness, have provided an appropriate chance to overcome the incompetence and discrepancies which may take place during conventional drug optimization and development techniques [16,17]. Machine learning (ML) is a predictive mathematical approach based on AI, which has paved the way to estimate the solubility of drugs in CO_2_SCF. To increase the generalization and performance of a single model, an ensemble of models is used in ML. Because of the blending of diverse predictions, ensembles generate effective predictive algorithms with enormous generalization capability [18]. Some ML techniques, such as decision tree and linear regression, are inherently unstable, which means that changing the training dataset results in a significantly different estimator. Unstable estimators have a low bias and high variability. Ensemble approaches have been proposed to reduce generalization error, that is, to reduce variance, bias, or both. In these approaches, the training dataset is modified, and an ensemble of different base estimators is created. These estimators are then combined to create a single estimator [19]. This section provides a quick overview of three main ensemble algorithms: bagging, gradient boosting, and Extra Trees.

In this study, a few basic models were first studied. Given that the decision tree gave significantly better outcomes, but these findings were not general enough to be presented as a powerful model backend, it was decided to employ models that reinforce it. Bagging and boosting are known as the most efficient advanced methods with decision trees. Bagging (bootstrap aggregating), created by Breiman et al. [20], can be considered as both a principal approach and a straight ensemble approach, illustrating brilliant efficiency as long as it reduces variance and avoids overfitting. The bootstrap technique, which replicates training datasets and creates training data subsets, contributes to the diversity of the bagging algorithm. Each subset is utilized to fit a various basic estimator, and the ultimate prediction outcomes are compiled by applying a majority vote procedure.

The other ensemble technique which is introduced from the work of Freund and Schapire is boosting [21]. In contrast to bagging, that provided a variety of basic learners by gradually reweighting the training data. Each sample weakly estimated by the previous estimator is given a higher weight in the next training step. As a result, training samples weakly estimated by predecessors are more likely to occur in the following bootstrap sample, and bias can be removed effectively. The final boosting algorithm model integrates all the underlying base estimators, which are weighted using their prediction performance.

There is a recently developed decision tree model called Extremely Random Tree (ExtraTree) that is an improved version of the traditional top-down decision tree (DT) model. There is an ensemble of DTs that has been trained in a recursive manner. The final model is built using a massive DT that is trained in a recursive manner. In each case, the tree must be expand using the entire set of data, and the proper cut point for each split can be calculated through the amount of data gained from each split [22,23].

The three algorithms selected for this study are:

- Random Forest (Bagging of Regression Trees);
- Extra Trees (Bagging of Regression Trees);
- Gradient Boosting (Boosting of Regression Trees).

## 2. Dataset

**Figure 1:**
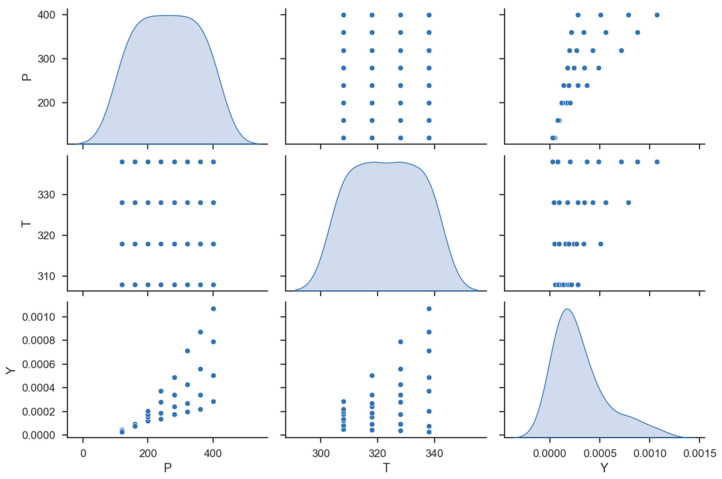
Data distribution, P (pressure), T (temperature), and Y (solubility).

Solubility models were created using a dataset with 32 input vectors, similar to [24]. The dataset is illustrated in Table 1. The distribution of features and output is shown in Figure 1. The diagonal subplots (when the x-axis and y-axis are identical) of this figure also show the kernel density estimate (KDE) plot. KDE plots visualize the distribution of observations in a dataset, like histograms. With KDEs, one or more dimensions of probability density curves are used to represent the data.

## 3. Methodology

### 3.1. Random Forest Regression (RFR)

This regression method is an ML procedure which estimates the targeted output by combining the results of several DT learners [25,26]. When Random Forest receives an *(x)* input data point, it includes the amounts of the various input features probed for a given training area, which creates *K* regression trees and the averages of their results. The RF regression predictor, after such *K*-trees {*T(x)*}*_1_^K^* have been trained, is [27]:

$${\hat{f}}_{rf}^{K}\left(x\right)=\frac{1}{K}\overset{K}{\underset{k=1}{\sum }}T\left(x\right)$$

For bypass tree correlation, Random Forest makes the trees grow using different training subsets generated across a routine called bagging. This process is a subset creation technique that involves resampling the original dataset randomly with replacement, in order to generate the next training subset {h (x, Θ_*k*_), *k* = 1, 2, …, *K*}, where each Θ_*k*_ is the same distributed independent random vector. Accordingly, some data can be applied many times during training, while some data points may never be used. By creating a tree using RF, the best split point will be created through a set of input light [28,29,30].

Moreover, the data which are not used in training step in the *k*-th tree model in the bagging method, they have been utilized in an out-of-bag subset (oob). *k*-th tree model be able to utilize the oob items to calculate accuracy [29]. Moreover, the non-selected items in the training step of the *k*-th tree along the bagging routine are considered in the out-of-bag subset (oob). On the other hand, the *k*-th tree must be able to utilize these oob items in order to evaluate the efficiency. Increasing the quantity of trees results in a reduction in error, which is illustrated by the fact that the Random Forest does not have an overfitting issue. The relative importance of the features is likewise determined using RF. In order to select the best features in multi-source investigations, it is critical to find the relationship between each item and predicted procedure, and this feature can assist with that understanding [30,31].

### 3.2. Extra Tree Regression (ETR)

Geurts et al. [32] introduced the extremely randomized tree (ExtraTree), which is an improved version of traditional top-down decision tree (DT) models. The ExtraTree is an ensemble of DTs that has been trained in a recursive manner, and the final model was built employing a massive DT. Each develops the tree utilizing the whole dataset, and the suitable cut point for each split can be decided through achieved information [22,23].

This model is very close to the Random Forest and the Extra Tree model’s primary innovations in that (i) the nodes are separated randomly when applying cut points, and (ii) the whole training dataset was used for developing the decision tree instead of subset generation using the bootstrap in Random Forest method [23,33].

### 3.3. Gradient Boosting Regression Trees (GBRT)

To improve prediction accuracy, boosting uses a series of base estimator compare to a single predictor to get an average between them. Base estimators/models (such as decision trees) are coordinated to clear bias in a stage-wise process. In order to modify the loss function, a new learner is introduced at each phase. Using training data, the first learner decreases the loss function to the lowest amount [34,35,36]. The following estimators make use of the previous estimators’ residuals. The following Algorithm 1 demonstrates the gradient boosting procedure [35,36,37]:
**Algorithm 1**Initialize $F_{0}\left(x\right)={\mathrm{argmin}}_{p}\sum_{i-1}^{N}L\left(y_{i},P\right)$ $\mathrm{For}\;m\;\in \left\{1,\;2,\;\ldots ,\;M\right\}:$   1. Compute the negative gradient ${\;\;\;\;\;\;\;\;\;\;\bar{y}}_{i}=-\left[\frac{\partial L\left(y_{i},F\left(x_{i}\right)\right)}{\partial F_{x_{i}}}\right]$   2. Create a model $\;\;\;\;\;\;\;\;\;\;a_{m}={\;\mathrm{argmin}}_{a,\beta }\overset{N}{\underset{i=1}{\sum }}{\left[\bar{y}-\beta h(x_{i},a_{m}\right]}^{2}$   3. Select a gradient descent step size as $\;\;\;\;\;\;\;\;\;\;p_{k}={\mathrm{argmin}}_{p}\overset{N}{\underset{i=1}{\sum }}L\left(y_{i},F_{m-1}\left(x_{i}\right)+\mathrm{ph}\left(x_{i},a\right)\right)$   4. Modify the estimation of F(x) $\;\;\;\;\;\;\;\;\;\;\;\;\;\;\;F_{m}\left(x\right)=F_{m-1}\left(x\right)+p_{k}h\left(x,a_{m}\right)$ Output: the aggregated regression function $F_{m}\left(x\right)$

Here, *x* demonstrates the feature vector and y demonstrates the corresponding class label. {x_i_, y_i_}N_i_ = 1,as training data and the aim is to calculate F_*_(x), be able to design x to y graph, a specific loss function L(y, F(x)) could be reduced to the lowest amount.

## 4. Results

After tweaking the hyper-parameters of models by testing various combinations of them, we employed *MAPE* and *R^2^* [38] to verify the accuracy and generality of the models.

The performance success of estimating findings is frequently measured using *R*-squared, which is without a doubt the most often utilized criterion. Using this metric, you can see how closely the predicted results match up with the observed data [39].

$$R^{2}=1-\frac{\sum {\left(e_{i}-o_{i}\right)}^{2}}{\sum {\left(o_{i}-{\;\bar{o}}_{i}\right)}^{2}}$$

**Figure 2:**
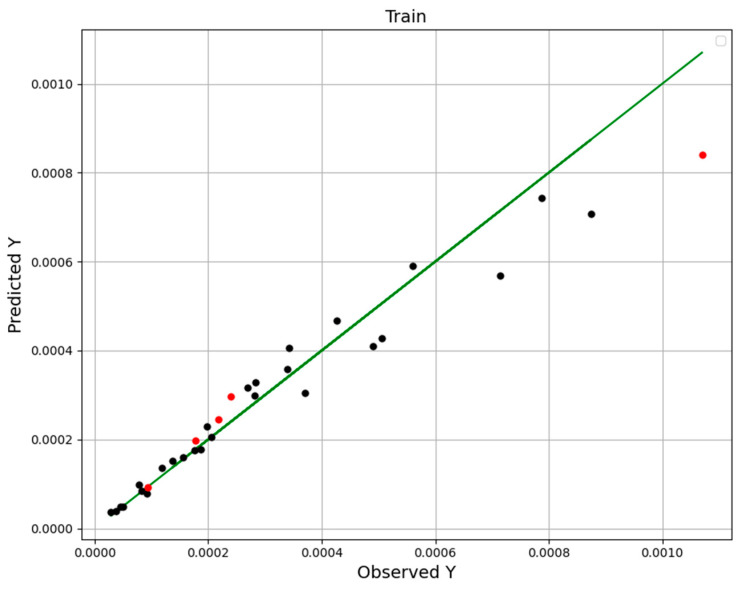
RFR Model: test and train data predictions.

**Figure 3:**
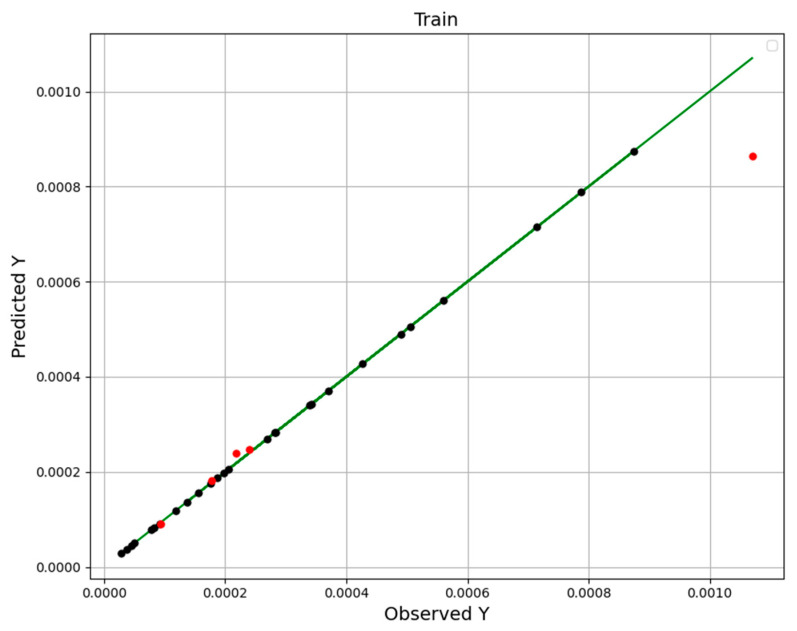
ETR Model: test and train data predictions.

**Figure 4:**
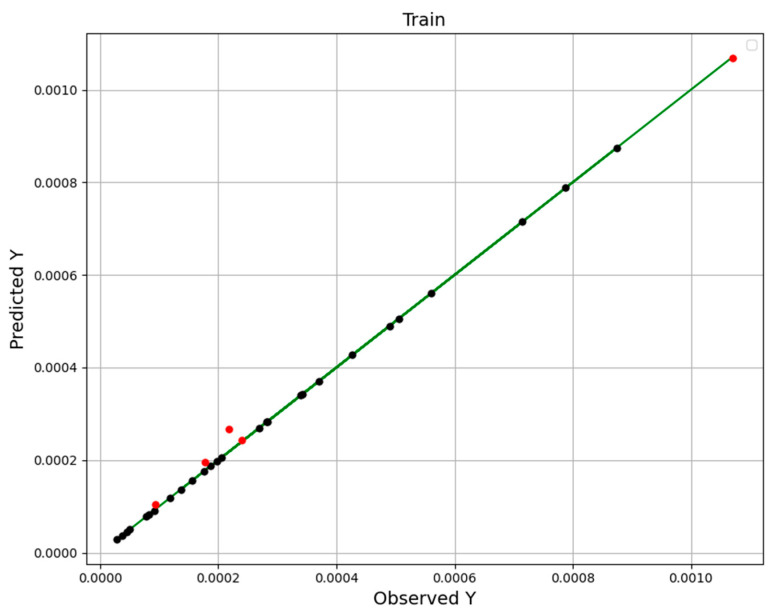
GBRT Model: test and train data predictions.

*MAPE* is also one of the most used evaluation metrics. *MAPE* illustrates error size, which is between 0 and 1 [40].

$$MAPE=\frac{1}{n}\overset{n}{\underset{i=1}{\sum }}|\frac{o_{i}-e_{i}}{o_{i}}|$$

e_i_ and *o_i_* are predicted and actual (observed) values. ${\bar{o}}_{i}$ is the average of the actual data. *n* indicates the size of the dataset. Comparisons of the actual and estimated values using RFR, ETR, and GBRT ML-based mathematical models are depicted in Figure 2, Figure 3 and Figure 4. In these figures, points are estimated amount (black is used as training and the other one, red can be used as the test) and the line indicates the actual values. The results imply that the GBRT method is the most general and precise model. Consideration of the *R^2^* and RMSE amounts through Table 2 confirms the greater accuracy of the GBRT model with the best generality.

The reason why GBRT is superior to the other two models is because the dataset is small. Therefore, every data point can have a profound impact on the final model. In order to improve air performance, it has been shown that the boosting method, by which the points that are incorrectly predicted are corrected by weighting, is more effective than the conventional method.

**Figure 5:**
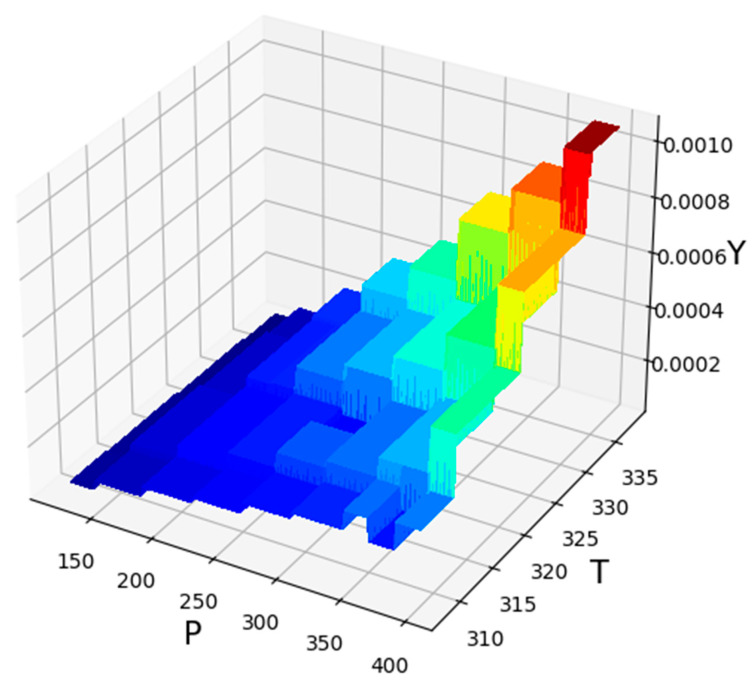
Three-dimensional illustration of inputs/outputs (GBRT Model).

**Figure 6:**
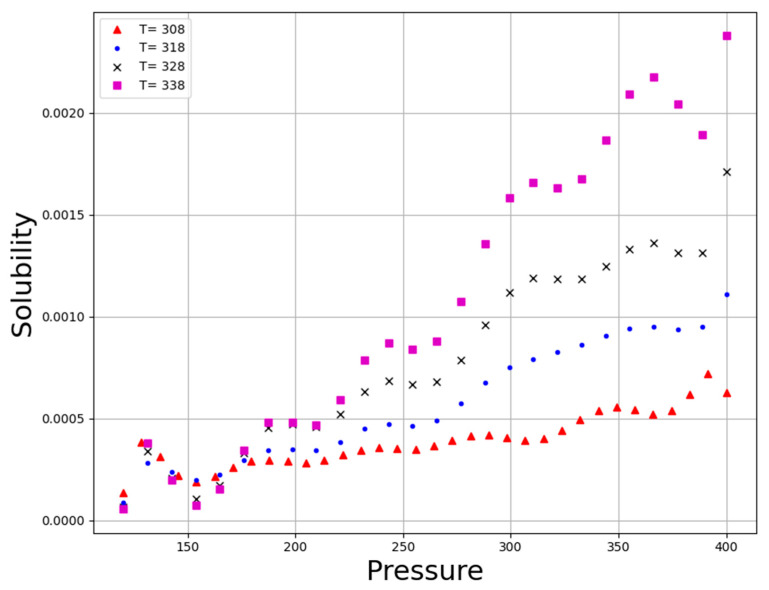
Tendency of P.

**Figure 7:**
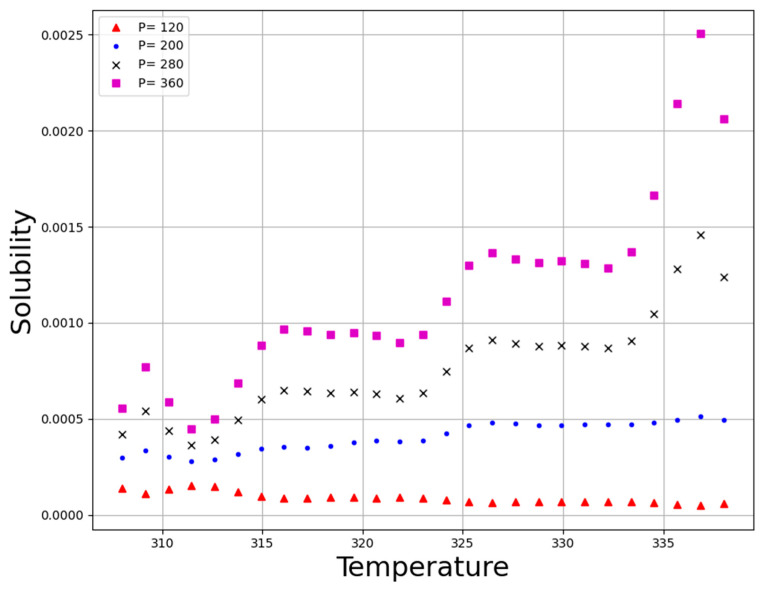
Tendency of T.

Figure 5 illustrates the three-dimensional result based on the GBRT predictive model to simultaneously evaluate the effect of those two parameters, pressure and temperature as input parameters on the solubility of the anti-cancer drug decitabine as the only output. Additionally, Figure 6 and Figure 7 schematically demonstrate two-dimensional variations in pressure and temperature versus decitabine solubility. For all evaluated isotherms, an increase in pressure considerably improves the density of CO_2_SCF due to an increase in the compaction of molecules. The increment in density resulted in an enhancement in the efficiency of the solvent and, therefore, the solubility value of decitabine in CO_2_SCF increases. Despite the correlation between pressure and drug solubility, the influence of temperature is not straightforward, and a reverse alteration is seen after the 200-bar pressure point. It is worth pointing out that the solvent density and the sublimation pressure are considered as two competing parameters which entirely affect the effect of temperature on the solubility of the drug. By increasing the temperature, the density of the solvent significantly reduces, since greater molecular energy eventuates in the free movement of solvent molecules. Moreover, enhancement in the temperature of the system can improve the sublimation pressure, which presents a positive influence on drug solubility in a supercritical system. Considering the description, the net effect of the abovementioned competing factors can determine the positive or negative role of temperature on drug solubility. Analysis of the graphs illustrate a threshold pressure, the temperature increment positively encourages drug solubility owing to the significant role of sublimation pressure in comparison to density. Further, in pressure below the cross-over pressure, an increase in the temperature results in a substantial decrement in the decitabine solubility because of the decline in the solvent density. According to Table 3, 380.88 bar and 333.01 K, can be mentioned as the optimum values at highest decitabine solubility.

## 5. Conclusions

In this study, various predictive models were developed using AI approaches to estimate the optimum solubility value of the anti-cancer drug decitabine inside carbon dioxide supercritical fluid (CO_2_SCF). We used three ensemble methods to build models on a small dataset: Random Forest (RFR), Extra Tree (ETR), and Gradient Boosted Regression Trees (GBRT). Various configurations were tested, and optimal hyper-parameters were discovered. The final models were then evaluated using industry-standard metrics. RFR, ETR, and GBRT all had R2 scores of 0.925, 0.999, and 0.999, respectively. Furthermore, the MAPE metric error rates were 1.423 × 10^−2^, 7.573 × 10^−2^, and 7.119 × 10^−2^, respectively. GBRT was selected as the primary method for this study through these facts and other visual considerations. The optimal values were calculated using this model as P = 380.88, T = 333.01, and Y = 0.001073.

## Figures and Tables

**Table 1:** The whole dataset.

No	X1 = P (Bar)	X2 = T (K)	Y = Solubility (Mole Fraction)
1	120	308	5.04 × 10^−5^
2	120	318	4.51 × 10^−5^
3	120	328	3.69 × 10^−5^
4	120	338	2.84 × 10^−5^
5	160	308	8.23 × 10^−5^
6	160	318	9.37 × 10^−5^
7	160	328	9.11 × 10^−5^
8	160	338	7.79 × 10^−5^
9	200	308	1.18 × 10^−4^
10	200	318	1.55 × 10^−4^
11	200	328	1.77 × 10^−4^
12	200	338	2.05 × 10^−4^
13	240	308	1.37 × 10^−4^
14	240	318	1.87 × 10^−4^
15	240	328	2.82 × 10^−4^
16	240	338	3.71 × 10^−4^
17	280	308	1.76 × 10^−4^
18	280	318	2.40 × 10^−4^
19	280	328	3.42 × 10^−4^
20	280	338	4.90 × 10^−4^
21	320	308	1.97 × 10^−4^
22	320	318	2.69 × 10^−4^
23	320	328	4.27 × 10^−4^
24	320	338	7.15 × 10^−4^
25	360	308	2.18 × 10^−4^
26	360	318	3.40 × 10^−4^
27	360	328	5.60 × 10^−4^
28	360	338	8.74 × 10^−4^
29	400	308	2.83 × 10^−4^
30	400	318	5.06 × 10^−4^
31	400	328	7.88 × 10^−4^
32	400	338	1.07 × 10^−3^

**Table 2:** Outputs.

Models	R^2^ Score	MAPE
RFR	0.925	1.423 × 10^−1^
ETR	0.999	7.573 × 10^−2^
GBRT	0.999	7.119 × 10^−2^

**Table 3:** Optimal values (GBRT Model).

P (Bar)	T (K)	Y
380.88	333.01	0.001073

## Data Availability

All data are available within the published paper.
